# Correlation between serum ANGPTL4 levels and white matter hyperintensity and cognitive impairment in patients with cerebral small vessel disease

**DOI:** 10.1002/brb3.3401

**Published:** 2024-02-04

**Authors:** Jianhua Zhao, Shiyun Zhang, Xiaoting Wang, Minghua Wang, Zehua Wang, Ruotai Li, Yuxi Liu, Chengbiao Lu, Shaomin Li

**Affiliations:** ^1^ Henan Joint International Research Laboratory of Neurorestoratology for Senile Dementia, Henan Key Laboratory of Neurorestoratology, Department of Neurology First Affiliated Hospital of Xinxiang Medical University Xinxiang Henan China; ^2^ Ann Romney Center for Neurologic Diseases, Brigham and Women's Hospital Harvard Medical School Boston Massachusetts USA; ^3^ Henan International Joint Laboratory of Non‐Invasive Neuromodulation, Department of Physiology and Neurobiology Xinxiang Medical University Xinxiang Henan China; ^4^ Department of Neurology Beijing Tiantan Hospital, Capital Medical University Beijing China

**Keywords:** ANGPTL4, cerebral small vessel disease, cognitive impairment, white matter hyperintensity

## Abstract

**Objectives:**

To investigate the correlation between serum angiopoietin‐like protein 4 (ANGPTL4) levels, white matter hyperintensity (WMH), and cognitive impairment (CI) in patients with cerebral small vessel disease (CSVD).

**Methods:**

This cross‐sectional study enrolled 171 patients with CSVD who attended the First Affiliated Hospital of Xinxiang Medical University from December 2021 to July 2022. All subjects underwent a 3.0T head magnetic resonance imaging, neuropsychology assessment, and blood sampling. Serum ANGPTL4 levels were detected by enzyme‐linked immunosorbent assay and the severity of WMH was assessed by the Fazekas scale. According to the Montreal Cognitive Assessment (MoCA) scale, subjects were divided into normal cognition group (NC, *n* = 80) and CI group (*n* = 91). According to the total Fazekas scores, subjects were divided into a mild WMH group (*n* = 84), a moderate WMH group (*n* = 70), and a severe WMH group (*n* = 17).

**Results:**

Serum ANGPTL4 levels were significantly higher in the CI group than in the NC group (*p* < .05) and were negatively correlated with mini–mental state examination scores and MoCA scores (*r* = −0.26, −0.341, *p* < .05). Serum ANGPTL4 levels increased significantly in the mild to moderate WMH group but tended to decrease in the severe WMH group. Binary logistic regression analysis showed that ANGPTL4 was an independent influencing factor for CSVD‐CI (OR = 2.062, 95% CI (1.591–2.674), *p* < .001). The area under curve of ANGPTL4 for CSVD‐CI was 0.847 (0.791–0.903).

**Conclusion:**

ANGPTL4 may be involved in the process of white matter damage and CI in CSVD patients and shows a diagnostic value for CSVD‐CI.

## Graphical abstract

ANGPTL4 is an independent influencing factor of CSVD‐CI after adjusting for other risk factors. ANGPTL4 levels increased gradually in mild to moderate WMH patients, but decreased in severe WMH patients. ANGPTL4 is expected to be a biomarker for the early identification of WMH and cognitive impairment progression in CSVD patients.

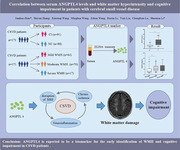

## 1 INTRODUCTION

Cerebral small vessel disease (CSVD) refers to a group of clinical, imaging, and pathological syndromes caused by various functional and structural lesions of cerebral small vessels (Zanon Zotin et al., 2021). Cognitive impairment (CI) is the most common clinical manifestation of CSVD. CSVD‐related CI (CSVD‐CI) may be caused by both vascular risk factors and neurodegeneration (Zanon Zotin et al., 2021). White matter hyperintensity (WMH) is the most common imaging marker of CSVD, either alone or in conjunction with other imaging markers, such as recent small subcortical infarcts, lacunar infarction of presumed vascular origin, enlarged perivascular space, and cerebral microbleed. WMH can cause disruption of functional connectivity and white matter microstructure in the default mode network of CSVD patients, leading to CI (Chen et al., 2019).

Angiopoietin‐like protein 4 (ANGPTL4), a member of the ANGPTLs family (ANGPTL1–ANGPTL8), is a secreted glycoprotein whose expression is regulated by fasting, metabolism, hypoxia, and other factors (Aryal et al., 2019). ANGPTL4 participates in the process of atherosclerosis by mediating the activity of lipoprotein lipase to regulate glucose and lipid metabolism (Aryal et al., 2019; Fernández‐Hernando & Suárez, 2020). In addition, ANGPTL4 is involved in the regulation of angiogenesis, vascular permeability, chronic inflammation, and hematopoietic stem cell proliferation (Fernández‐Hernando & Suárez, 2020; Zheng et al., 2021).

Previous studies have demonstrated that ANGPTL4 can be expressed in coronary heart disease (CHD) (Katanasaka et al., 2022), hypertension (Abu‐Farha et al., 2018), and ischemic stroke (Zheng et al., 2021). Studies also showed that ANGPTL4 may play a role in the pathogenesis of neurodegenerative disease. The ANGPTL4 gene was upregulated in patients with Alzheimer's disease (AD) and CSVD (Leandro et al., 2018; Ritz et al., 2017). Chakraborty et al. (2018) found that plasma ANGPTL4 levels were elevated in patients with AD and vascular dementia. There are few studies investigating the relationship between ANGPTL4 and WMH in CSVD patients, the levels of ANGPTL4 in patients with CSVD‐CI are unclear. This study elucidated the relationship between ANGPTL4, WMH, and CI in CSVD patients.

## 2 MATERIALS AND METHODS

### 2.1 Participants

A total of 171 CSVD patients were enrolled who were admitted to the First Affiliated Hospital of Xinxiang Medical University from December 2021 to July 2022, with complaints of “dizziness, headache, memory impairment, and unsteadiness in walking, etc.” Inclusion criteria: (1) 40–80 years; (2) met the diagnostic criteria of CSVD (Wardlaw et al., 2013); (3) with mild to severe WMH; (4) independence in daily life (modified Rankin scale ≤2); (5) complete imaging and neuropsychological data, as defined below. Exclusion criteria: (1) acute cerebral infarction lesions >20 mm in diameter, acute intracranial hemorrhagic disease, intracranial and extracranial large vessel stenosis (>50% stenosis); (2) nonvascular factors such as metabolic, toxic, infectious, genetic, tumor, and other causes of white matter lesions; (3) other neurological disorders, such as Parkinson's disease, AD, multiple sclerosis, neuromyelitis optica spectrum disorders, epilepsy, and dementia; (4) other diseases affecting cognitive function, such as disorders of consciousness, aphasia, mental retardation, psychiatric disorders, and cranial trauma; (5) systemic diseases, such as shock, severe cardiopulmonary and renal diseases, hematology, infections, and rheumatic immune system diseases; (6) recent use of hormones, antibiotics, nootropic drugs, antipsychotics, and other drugs that affect outcomes.

This study was approved by the Ethics Committee of the First Affiliated Hospital of Xinxiang Medical University (approval number: 2020018), and all patients signed the informed consent form.

### 2.2 Data collection

Demographic information (age, gender, and education), past history (hypertension, diabetes mellitus, hyperlipidemia, stroke, CHD, smoking, and alcohol use), and past medication history (lipid‐regulating drugs, angiotensin‐converting enzyme inhibitors [ACEI], or angiotensin receptor blockers [ARB]) were collected from all CSVD patients. Biochemical parameters, such as total cholesterol, triglycerides, high‐density lipoprotein, low‐density lipoprotein, fasting blood glucose, hemoglobin A1c (HbA1c), uric acid, and homocysteine (Hcy), were collected.

### 2.3 Determination of ANGPTL4 levels

All patients had 5 mL of fasting elbow venous blood drawn within 24 h of admission, placed in non‐anticoagulant blood collection tubes, left at room temperature for 15 min, centrifuged at 3000 r/min for 10 min, and the upper layer of serum was carefully separated and placed in −80°C refrigerator to be frozen for use. Serum ANGPTL4 levels were detected by enzyme‐linked immunosorbent assay (ELISA). The procedure was performed according to the instruction of the ANGPTL4 ELISA Kit (EH0038, Wuhan Feien Biotechnology Co., Ltd.).

### 2.4 Neuropsychological assessment

The Mini–Mental State Examination (MMSE) and the Montreal Cognitive Assessment (MoCA) scales were jointly evaluated in the same environment using uniform instructions by two trained neurologists, and all patients completed within 1 week after admission. The MoCA scale was used to evaluate damage of different cognitive domains, including visuospatial space and executive function (5 points), naming (3 points), attention (6 points), language (3 points), abstract (2 points), memory (5 points), and orientation (6 points). Based on a study of cognitive function in Chinese population, total MoCA scores ≤13 for illiterates, ≤19 for those with 1–6 years of education, and ≤24 for those with 7 or more years of education were defined as CI (Lu et al., 2011).

According to the “Chinese Clinical Guidelines for Cerebral Small Vessel Disease‐Related Cognitive Impairment 2019” (Peng, 2019) and MOCA score adjusted according to education, 171 CSVD patients were divided into normal cognition (NC) group (*n* = 80) and CI group (*n* = 91).

### 2.5 Neuroimaging assessment

**FIGURE 1:**
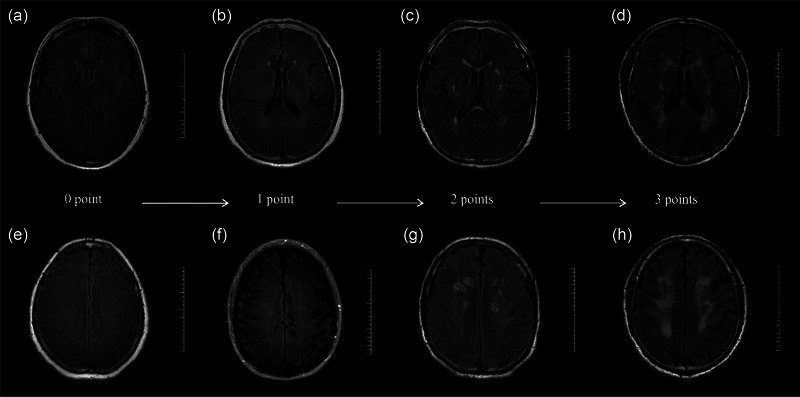
Magnetic resonance imaging (MRI) imaging of cerebral small vessel disease (CSVD) patients with different severity of white matter hyperintensity (WMH) in T2FLAIR. Periventricular WMH (PWMH): (A) (0 point), (B) (1 point), (C) (2 points), and (D) (3 points); deep WMH (DWMH): (E) (0 point), (F) (1 point), (G) (2 points), and (H) (3 points).

All CSVD patients underwent brain magnetic resonance imaging (MRI) using a 3.0T MRI scanner (GE) with an 8‐channel head coil, including conventional axial T1WI, T2WI, T2FLAIR, and DWI sequences. The scanning parameters as shown in the Supplementary material. Two neuroradiologists assessed independently the severity of WMH using the Fazekas scale single‐blind to clinical symptoms (Fazekas et al., 1987). Periventricular WMH (PWMH) and deep WMH (DWMH) were scored separately, and the two components were summed to calculate the total score. PWMH scores: (1) 0 point: no lesion; (2) 1 point: thin cap or pencil‐like lesion; (3) 2 points: smooth halo; (4) 3 points: irregular periventricular WMH extending into the deep white matter. DWMH: (1) 0 point: no lesion; (2) 1 point: punctate lesion; (3) 2 points: lesion begins to fuse; (4) 3 points: extensive fusion of lesions. The total Fazekas scores reflect WMH load, as shown in Figure 1. Total Fazekas scores of 1 and 2 points are defined as mild WMH group (*n* = 84), 3 and 4 points as moderate (*n* = 70), and 5 and 6 points as severe (*n* = 17).

### 2.6 Statistical analysis

All data were statistically analyzed by SPSS version 25.0 (IBM Corp.). Continuous variables conforming to normal distribution were described by mean ± standard deviation, and an independent sample *t*‐test was used for comparison between two groups. Continuous variables of skewness distribution were represented by median (*P*25, *P*75), and a comparison between two groups was performed by Mann–Whitney *U* test. ANGPTL4 levels were compared among the mild WMH, moderate WMH, and severe WMH groups using Bonferroni test in a two‐way comparison. Categorical variables were expressed as percentages, and comparison between groups was conducted using the chi‐square test or Fisher exact test. Binary logistic regression was used to analyze the factors that might affect CSVD‐CI. Correlation of ANGPTL4 with WMH and CI by Spearman correlation analysis. To analyze the diagnostic value of ANGPTL4 for CSVD‐CI by plotting the receiver operating characteristic (ROC) curve. *p* < .05 was considered statistically significant.

## 3 RESULTS

### 3.1 Baseline information

The demographic and clinical characteristics of 171 CSVD patients were shown in Table 1, with an average age of (61.22 ± 8.53) years, and 56.1% were male. There were statistical significance in age, education, serum Hcy, and HbA1c between the two groups (*p* < .05). There were no statistical significance in gender, body mass index, history of smoking, alcohol use, hypertension, diabetes mellitus, history of stroke, history of CHD, history of lipid‐regulating drugs, and history of ACEI or ARB drugs (*p* > .05).

**TABLE 1:** Demographic information, biochemical indicators between two groups.

Variables	NC (*n* = 80)	CI (*n* = 91)	*t*/*χ* ^2^/u	*p*
Age, years	58.93 ± 8.09	63.24 ± 8.44	−3.402	**.001**
Sex (male)	43 (53.8)	53 (58.2)	.349	.555
Education, years	8 (5, 9)	7 (4, 8)	−2.494	**.013**
BMI, kg/m^2^	25.39 ± 3.49	25.35 ± 3.88	.075	.94
Smoking history	25 (31.3)	28 (30.8)	.005	.946
Alcohol use	16 (20)	17 (18.7)	.048	.827
Hypertension	58 (72.5)	72 (79.1)	1.024	.312
Diabetes mellitus	17 (21.3)	26 (28.6)	1.212	.271
CHD	8 (10)	9 (9.9)	.001	.981
History of stroke	23 (28.7)	35 (38.5)	1.791	.181
History of lipid‐regulating drugs	26 (32.5)	33 (36.3)	.267	.605
History of ACEI or ARB drugs	21 (26.3)	25 (27.5)	.032	.857
TC, mmol/L	4.27 (3.55, 5.15)	4.22 (3.48, 5.08)	−.906	.365
TG, mmol/L	1.27 (0.92, 1.86)	1.17 (0.84, 1.65)	−1.371	.17
LDL, mmol/L	2.61 ± 0.93	2.4 ± 0.85	1.527	.129
HDL, mmol/L	1.11 (0.96, 1.35)	1.16 (1.05, 1.35)	−.615	.539
FBG, mmol/L	5.15 (4.7, 5.88)	5.01 (4.64, 6.28)	−.271	.786
HbA1c, %	5.71 (5.34, 6)	5.9 (5.56, 6.12)	−2.166	**.03**
Hcy, umol/L	13.72 (11.04, 18.47)	14.8 (12.58, 21.88)	−1.977	**.046**
Cr, umol/L	61.45 (50.85, 68.48)	64.1 (53.2, 72)	−1.757	.079
SUA, umol/L	266 (225.25, 314.25)	272 (223, 327)	−.24	.81

*Note*: Data were presented as mean ± SD or median (*P*25, *P*75) or *n* (%).

Abbreviations: ACEI, angiotensin‐converting enzyme inhibitors; ARB, angiotensin receptor blockers; BMI, body mass index; CHD, coronary heart disease; Cr, creatinine; CI, cognitive impairment; FBG, fasting blood glucose; HbA1c, hemoglobin A1c; Hcy, homocysteine; HDL, high‐density lipoprotein; LDL, low‐density lipoprotein; NC, normal cognition; SUA, serum uric acid; TC, total cholesterol; TG, triglyceride.

### 3.2 Correlation between WMH and cognitive impairment

Mild WMH accounted for 28.6%, moderate WMH accounted for 53.8%, and severe WMH was 17.6% in the CI group, as shown in Table 2. There were statistical difference between the two groups in total Fazekas, PWMH, and DWMH scores (*p* < .001). Total Fazekas, PWMH, and DWMH scores were negatively correlated with MoCA scores (*r* = −.329, −.327, −.281, *p* < .05).

**TABLE 2:** Two groups of imaging indicators.

Imaging markers	NC (*n* = 80)	CI (*n* = 91)	*χ* ^2^	*p*
WMH			36.067	**<.001**
Mild (1–2 points)	58 (72.5)	26 (28.6)		
Moderate (3–4 points)	21 (26.3)	49 (53.8)		
Severe (5–6 points)	1 (1.3)	16 (17.6)		
PWMH/point			31.594	**<.001**
1	56 (70)	27 (29.7)		
2	23 (28.7)	48 (52.7)		
3	1 (1.3)	16 (17.6)		
DWMH/point			38.423	**<.001**
0	31 (38.8)	9 (9.9)		
1	44 (55)	45 (49.5)		
2	5 (6.3)	29 (31.9)		
3	0 (0)	8 (8.8)		
With other imaging markers of CSVD	38(47.5)	58 (63.7)	4.558	**.033**

*Note*: Data were presented as *n* (%).

Abbreviations: CI, cognitive impairment; CSVD, cerebral small vessel disease; DWMH, deep white matter hyperintensity; PWMH, periventricular white matter hyperintensity; NC, normal cognition; WMH, white matter hyperintensity.

### 3.3 Correlation between ANGPTL4 and cognitive function

**FIGURE 2:**
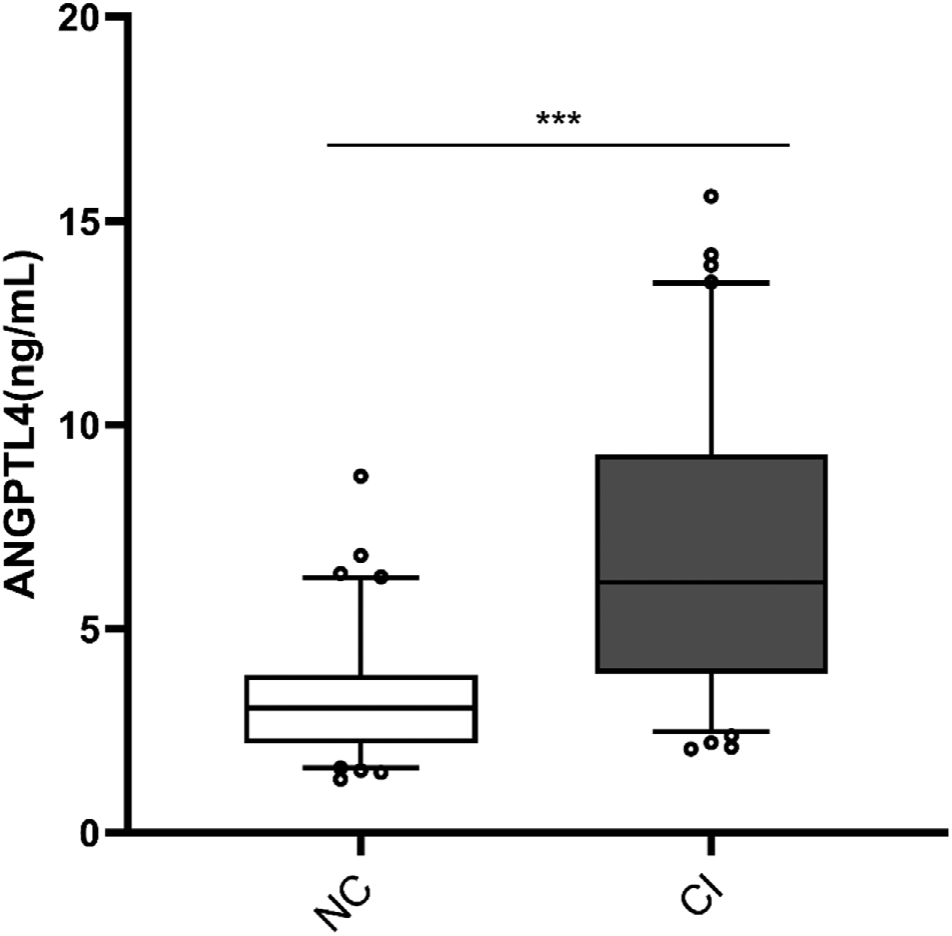
Box plots for serum angiopoietin‐like protein 4 (ANGPTL4) levels in cognitive impairment (CI) and normal cognition (NC) groups. Data are median (central line), interquartile range (box margins), and 5th and 95th percentile (whiskers). ***p < .001.

The median ANGPTL4 levels of 6.14 (3.9, 9.27) ng/mL in the CI group were significantly higher than that of 3.05 (2.18, 3.87) ng/mL in the NC group, with statistical significance (*p* < .001), as shown in Figure 2. According to Spearman's correlation analysis, ANGPTL4 levels were negatively correlated with MMSE and MoCA scores (*r* = −.26, −.341, *p* < .05), and ANGPTL4 levels were negatively correlated with MoCA subscales of visuospatial space and executive function, naming, attention, language, and memory (*r* = −.274, −.24, −.266, −.257, −.32, *p* < .05). As shown in Table 3.

**TABLE 3:** Correlation between angiopoietin‐like protein 4 (ANGPTL4) and cognitive function and white matter hyperintensity (WMH).

Variables	Score	ANGPTL4 (ng/mL)	
		*R*	*p*
MMSE	27 (25, 28)	−.26	.001
MoCA	21 (17, 25)	−.341	<.001
Visuospatial space and executive function	3 (2, 4)	−.274	<.001
Naming	3 (2, 3)	−.24	.002
Attention	6 (5, 6)	−.266	<.001
Language	2 (1, 2)	−.257	.001
Abstraction	2 (1, 2)	−.113	.142
Memory	1 (0, 2)	−.32	<.001
Orientation	6 (5, 6)	−.144	.061
Total Fazekas scores	3 (2, 3)	.316	<.001
PWMH	2 (1, 2)	.246	.001
DWMH	1 (1, 1)	.320	<.001

*Note*: Data were presented as median (*P*25, *P*75).

Abbreviations: DWMH, deep white matter hyperintensity; MMSE, mini–mental state examination; MoCA, montreal cognitive assessment; PWMH, periventricular white matter hyperintensity.

### 3.4 Correlation with ANGPTL4 and the severity of WMH

**FIGURE 3:**
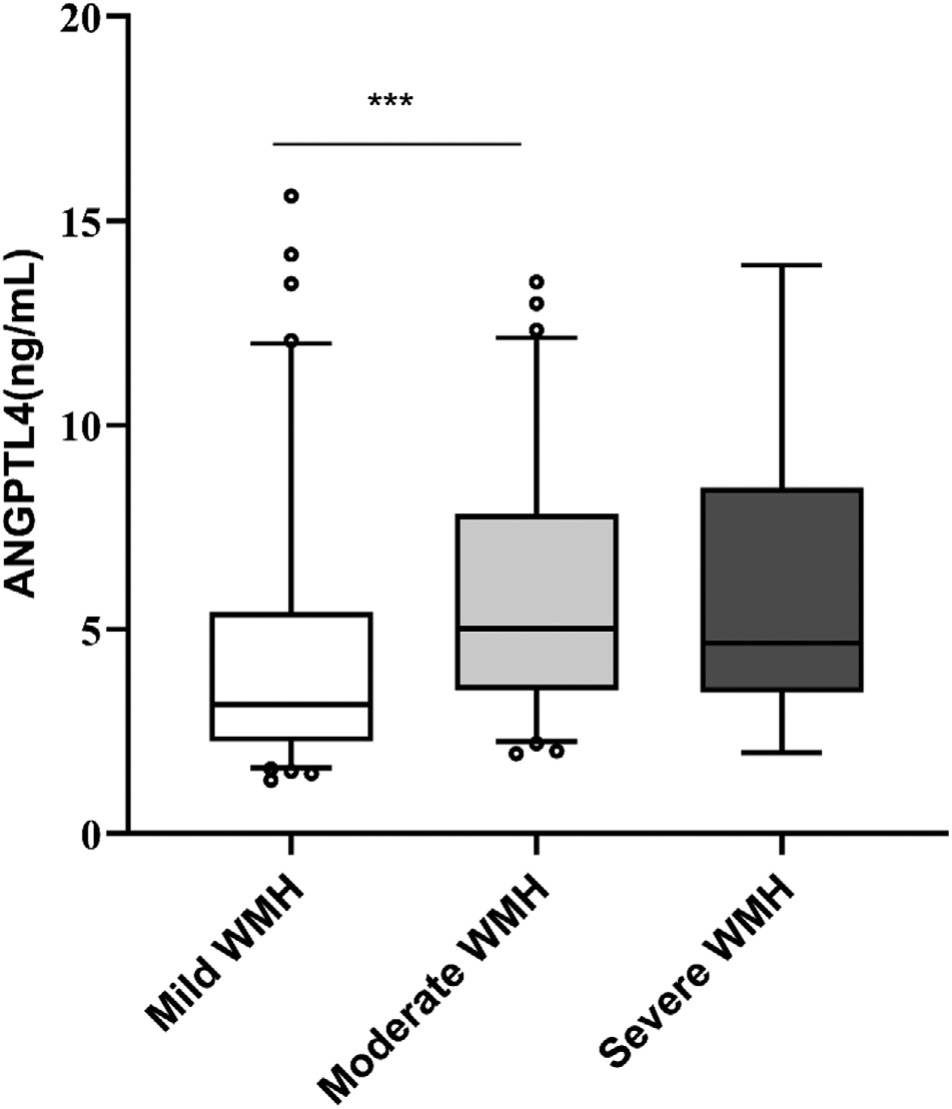
Box plots for serum angiopoietin‐like protein 4 (ANGPTL4) levels in different severity of white matter hyperintensity (WMH). Data are median (central line), interquartile range (box margins), and 5th and 95th percentile (whiskers). ***p < .001.

ANGPTL4 levels in the mild WMH group were lower than the moderate WMH group, with a statistical significance between the two groups (*p* < .001), as shown in Figure 3. Compared with the moderate WMH group, ANGPTL4 levels gradually decreased in the severe WMH group, with no statistical significance (*p* > .05). ANGPTL4 levels were positively correlated with total Fazekas, PWMH, and DWMH scores according to Spearman's correlation analysis (*r* = .316, .246, .32, *p* < .05). As shown in Table 3.

### 3.5 Multifactorial analysis of CSVD‐CI

**FIGURE 4:**
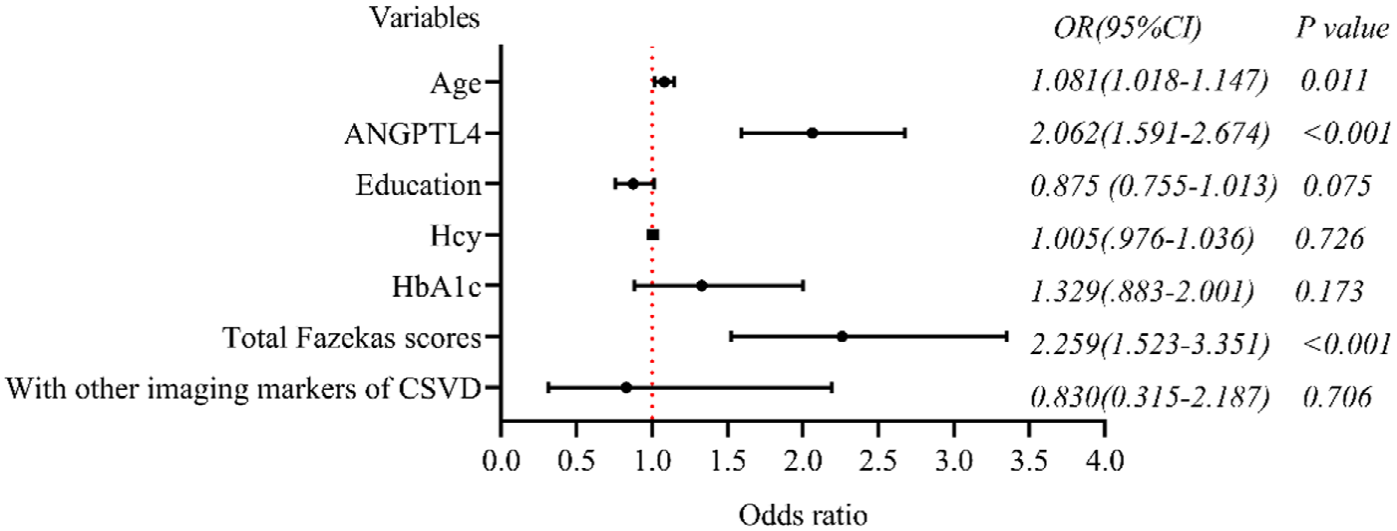
Logistic regression analysis of cognitive impairment (CI) in patients with cerebral small vessel disease(CSVD).

Binary logistic regression analysis was performed with the occurrence of CI as the dependent variable, age, education, ANGPTL4 levels, serum Hcy, HbA1c, total Fazekas scores, and other CSVD imaging markers as covariates. After adjusting other factors, ANGPLT4 was an independent influencing factor of CSVD‐CI (OR = 2.062, 95% CI (1.591–2.674), *p* < .001). As shown in Figure 4.

### 3.6 Diagnostic value of ANGPTL4 for CSVD‐CI

**FIGURE 5:**
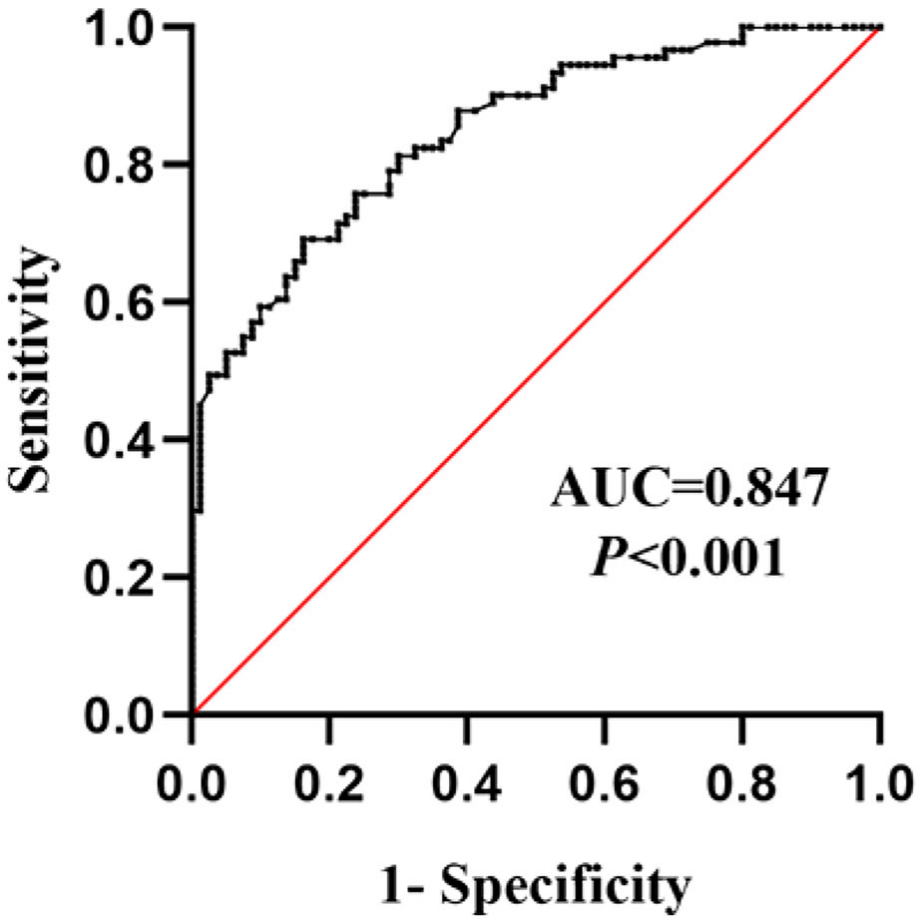
Receiver operating characteristic (ROC) curve of cognitive impairment (CI) in patients with cerebral small vessel disease (CSVD) diagnosed by ANGPTL4.

The ROC curve was used to analyze the diagnostic value of ANGPTL4 for CSVD‐CI, the area under curve was 0.847 (0.719–0.903), the optimal critical value was 4.54 ng/mL, the sensitivity was 0.692, and the specificity was 0.838, as shown in Figure 5.

## 4 DISCUSSION

This cross‐sectional study investigated the relationship between serum ANGPTL4 levels and WMH, and CI in patients with CSVD, suggesting ANGPTL4 may be involved in the process of white matter damage and CI in CSVD patients, and shows a diagnostic value for CSVD‐CI.

Currently, the clinical diagnosis of CSVD‐CI is mainly based on imaging markers and scale scores, whereas biomarkers have become a major avenue to explore the pathogenesis of CSVD in order to predict and treat CSVD‐CI in the early stage. This study found that ANGPTL4 was an independent influencing factor of CSVD‐CI and was negatively correlated with MMSE scores, which was consistent with previous research results (Chakraborty et al., 2018). A meta‐analysis involving 69 studies showed that CSVD could cause damage to all cognitive domains and found that low education level was a modifiable risk factor for CSVD‐CI (Hamilton et al., 2021), which was consistent with our research findings. Our finding revealed that serum ANGPTL4 levels were associated with impaired visuospatial space and executive functions, naming, attention, language, and memory. Therefore, it was hypothesized that ANGPTL4 may be a blood biomarker of CSVD‐CI. Serum ANGPTL4 levels were positively correlated with total Fazekas, PWMH, and DWMH scores, suggesting that ANGPTL4 may be involved in the process of white matter damage in CSVD patients.

WMH is a predictor of stroke, CI, dementia, and death and can be used as an intermediate marker of cerebrovascular events (Debette & Markus, 2010). More and more studies have shown that WMH can cause CSVD‐CI, WMH is associated with impaired subjective cognition, social cognition, attention, memory, executive function, and so on (Kynast et al., 2018; Xia et al., 2020). Our finding was consistent with a large French community cohort study of CSVD (*n* = 849), which showed a gradual decline in cognitive function as the severity of WMH increased (Kynast et al., 2018). It has been found that WMH and CSVD‐CI share the same pathogenesis, such as chronic ischemic hypoperfusion, disruption of blood–brain barrier, and chronic inflammation (Kerkhofs et al., 2021; Meng et al., 2019; Zanon Zotin et al., 2021), and that ANGPTL4 may play a dual role in these processes (Fernández‐Hernando & Suárez, 2020).

Research shows that ANGPTL4 is a hypoxia induced angiogenic factor (Babapoor‐Farrokhran et al., 2015). An autopsy study showed that increased ANGPTL4 expression was associated with increased cerebral microvessel density in patients with cerebral amyloid angiopathy, a phenomenon that may be caused by hypoxia (Chakraborty et al., 2018). Animal experiments also revealed that ANGPTL4 promotes neovascularization in ischemic stroke mice (Bouleti et al., 2013; Qiu et al., 2021). In an animal model of CSVD mice, chronic hypoxia induces increased expression of angiogenic factors, which promotes white matter neovascularization, remodeling, and white matter repair (Yang et al., 2018). We speculate that the possible reason for the elevated expression of ANGPTL4 levels in the CI group and the mild–moderate WMH group is that ANGPTL4 promotes white matter angiogenesis and thus improves cognitive function.

However, some studies have shown that ANGPTL4 has antiangiogenic effects (Okochi‐Takada et al., 2014; Yang et al., 2008). Whole cerebral vascular damage can be observed in animal models of CSVD, especially extensive small vessel reduction in white matter, which leads to white matter damage and CSVD‐CI (Wu et al., 2023). Lower angiogenic activity was related to higher WMH load in patients with mild CI and AD, and the decline of angiogenic activity was the first step leading to neuronal death and CI (Callahan et al., 2020). ANGPTL4 expression was reduced in the severe WMH group, possibly due to the small sample size, or possibly due to the fact that ANGPTL4 expression played an antiangiogenic role when reached to a certain extent in white matter damage, exacerbating CI.

There were some limitations in this study. First, it was a cross‐sectional study, which can only showed that ANGPTL4 was an independent influencing factor of CSVD‐CI and could not clarify its causal relationship with WMH and CSVD‐CI; second, the population included in this study was relatively small, the multicenter and large sample cohort studies are needed in the future. Meanwhile, animal experiments are needed to explore the mechanism of ANGPTL4 and white matter damage and CSVD‐CI in the further.

## 5 CONCLUSION

This study showed that ANGPTL4 was an independent influencing factor of CSVD‐CI. ANGPTL4 may be involved in the process of white matter damage and CSVD‐CI, whether its elevated expression is a protective factor or an impairing effect needs to be verified in animal experiments. In the future, ANGPTL4 may be a biomarker for the early identification of WMH and CSVD‐CI.

## AUTHOR CONTRIBUTIONS

*Conceptualization; formal analysis; funding acquisition; writing—review and editing*: Jianhua Zhao. *Conceptualization; data curation; investigation; methodology; writing—original draft*: Shiyun Zhang. *Data curation; visualization*: Xiaoting Wang. *Methodology; validation*: Minghua Wang. *Investigation; software*: Zehua Wang. *Methodology; formal analysis*: Ruotai Li. *Software; visualization*: Yuxi Liu. *Project administration; resources; supervision*: Chengbiao Lu. *Supervision; validation; writing—review and editing*: Shaomin Li. All authors contributed to the article and approved the submitted version.

## CONFLICT OF INTEREST STATEMENT

The authors declare no conflicts of interest.

## FUNDING INFORMATION

National Natural Science Foundation of China, Grant Number: 81771517; Natural Science Foundation of Henan Province, Grant Number: 182300410389; International Cooperation Project in Science and Technology of Henan Province, Grant Number: 232102521029

### PEER REVIEW

The peer review history for this article is available at https://publons.com/publon/10.1002/brb3.3401

## Supporting information

Supporting Information

## Data Availability

The datasets are available from the corresponding author upon reasonable request.
