# Supplementary material for: Correlation between serum ANGPTL4 levels and white matter hyperintensity and cognitive impairment in patients with cerebral small vessel disease
Source: Brain Behav. 2024 Feb 4;14(2):e3401. doi: 10.1002/brb3.3401 (PMC10839130; doi:10.1002/brb3.3401)
Supplement: Supplementary file 1 — Supporting Information [file BRB3-14-e3401-s001.docx]

Table S1. Parameters for conventional MRI sequences.

| Sequences | T1WI | T2WI | T2FLAIR | DWI |
| --- | --- | --- | --- | --- |
| TR, ms | 1750 | 3805 | 8400 | 3543 |
| TE, ms | Min full | 110 | 120 | Minimum |
| Slice spacing, mm | 1.5 | 1.5 | 1.5 | 1.5 |
| Slice thickness, mm | 5 | 5 | 5 | 5 |
| Matrix size | 320 × 192 | 352× 352 | 320× 192 | 162 × 192 |

Abbreviations: T1WI = T1-weighted imaging; T2WI = T2-weighted imaging; T2FLAIR = fluid-attenuated inversion recovery; DWI = diffusion-weighted imaging; TR = repetition time; TE = echo time.
